# Obstetrical Amenities

**Published:** 1906-07

**Authors:** Stephen Y. Howell

**Affiliations:** Buffalo, N. Y.; Surgeon to the Sisters of Charity, Mercy and Emergency Hospitals


### Obstetrical Amenities.

By STEPHEN Y. HOWELL, A. M., M. D., M. R. C. S. Eng., Buffalo, N. Y.,
Surgeon to the Sisters of Charity, Mercy and Emergency Hospitals.

OUR purpose in writing this paper is, briefly to review certain points pertaining to the active exercise of the obstetrical art, and having much to do with the physician's mental and physical comfort or discomfort. We shall consider not merely the pleasing features of the work,—the "amenities," as heralded in our title,—but its amaritudes as well. By thus properly blending the bitter with the pleasant, the amaritudes with the amenities, we round out our subject, as it were, and enhance the potential worth of our final inductions.

In the first place, it will be well to consider the wage of the obstetrician. Is it at all commensurate with the service rendered? Reflect for a moment upon the obligations assumed when engaged to see a woman safely through her period of travail. The physician does not simply agree to exercise his best knowledge and skill in his patient's behalf, but he obligates himself to attend personally to her needs. She engages her professional adviser, as a rule, because she believes him to be thoroughly competent and trustworthy. She wants *him* and none other. Were he to fail her in the hour of need, her woes are indeed multiplied. She is forced to rely upon the services of a substitute more or less unfamiliar to her. Though previously agreed upon, perhaps, the alternate is generally accepted with the hope, expressed or implied, that his assistance may not be required. Should even this second choice be denied her by reason of her physician's thoughtlessness, she is obliged, at the last moment, to confide herself to the ministrations of any practitioner who may be available. This last defection on the part of a medical attendant is bitterly resented, and rightfully so. Strong indeed is his position in the family, should the offense be subsequently condoned.

Considerations such as these obligate the strict fulfilment of the accoucheur's contract whenever this is possible. For days and weeks preceding the estimated date of parturition, indeed while the uterus still harbors its occupant, the doctor should be within call, lest he be found wanting in the hour of need. Concede the practical truth of this proposition, as indeed you must, and you concede that the obstetrician who possesses even a fair practice is a slave to duty. Absence is fraught with danger, vacations spell potential disaster.

It has been suggested that the practitioners of some future day will lessen the handicaps of the present by the artificial induction of labor. The proposition certainly has its attractive features.

The period for safe termination of gestation being determined, the obstetrician will appoint a convenient date for the initial steps, just as does the modern surgeon. Timely work could thus insure a day-time delivery with all its obvious advantages. Rested and wide-awake, the professional attendant would be prepared to empty the uterus secundum artem. Any necessary assistance could readily be obtained, and the theater of work best adapted to the needs of the individual case could be selected.

At present, however, experience and precept teach us that, after the inception of labor, we should rely largely upon Dame Nature. Of couse, the temptation to butt-in is terrible. Particularly is this true when the attendant is young, ignorant and correspondingly self-sufficient. Or, business appointments may be so exigent, and old lady Nature so tarnal slow, that even the older and more experienced are prone to excuse the so-called judicious use of gaff and spur.

From time immemorial, however, the loss of time incident to the care of the parturient has occupied a prominent place in the list of professional grievances; and, let me add, it will always, in all human probability, be thus exalted. Why? For the simple reason that until, in the course of man's evolution, the mechanics of labor are altered to that end, delay will be a necessary sequence.

Between the unavoidable and the useless loss of time, however, lies the broad field which separates the really capable, efficient attendant from the opposite type. The competent accoucheur is well informed and well trained. All the problems involved in the parturient act have been studied and pondered over to such a degree that he acts properly and intelligently. In the first place, he is careful to ascertain the precise conditions which confront him. He determines the position of the child and the circumstances which may affect its passage through the genital canal. The fetal pulse is located and, thereafter, serves as a monitor and guide, Such a physician has regard for the vitality of tissues, appreciates the disastrous consequences of too prolonged topical pressure upon the maternal structures, and actively interferes only to promote the welfare of mother or infant.

How painfully different is the conduct of the incompetent! Ignorant, timid, or both, he goes his way "by guess and by gosh", as does a blind guide. His ideas regarding the precise conditions which obtain are nebulous in the extreme. Like Mr. Micawber, of blessed memory, he waits fatuously for "something to turn up", hoping and praying that the "something" may prove to be the longed-for baby. He invades the aseptic passages of the mother with hands so richly provided with bacterial flora that he would obtain a prize in any laboratory devoted to the study of microbic parasites. Instruments are misused to the detriment of mother and child. Resultant lesions are overlooked, deliberately neglected, or repaired in a slovenly, unscientific manner. Such an attendant is far from being the peer of an intelligent midwife. And yet, wonder of wonders! should his patient survive, and despite the possible death of the child, he is only too often rewarded by the plaudits of an admiring audience. "'Twas a terrible case", exclaim they, "but the doctor pulled the mother through safely!"

The competent attendant, per contra, may be required to deal with conditions of the most serious and trying character. Alert, prompt, and inspired by the confidence begotten of knowledge, he surmounts all vincible difficulties and achieves the best possible outcome. Is his work appreciated? Hardly, as a rule. Such good things are taken for granted, received as a natural right. This very preversion of justice it is which constitutes the chief raison d'etre of professional incompetence. Pari passu, with the diffusion of knowledge among the masses, however, the indolent and ignorant practitioner will find his field of pernicious activity more and more circumscribed. He will eventually shrink to an intangible memory,—a historical has-been.

Our one excuse for criticism, seemingly so harsh, is the desire to recall most forcibly the importance of obstetrics as a branch of our art, the difficulties involved in its practice and the enormous responsibilities assumed by him who engages in the work. In our opinion, the trials of the accoucheur are tantamount to those of the surgeon. Loss of sleep, mental and physical fatigue, the difficulty of securing assistance in the night-season, the clamor and supererogatory advice of anxious friends and relatives, the responsibility incident to the care of two lives and the choice of procedure,—all these tend to paralyse one's efficiency.

In one case the writer was summoned to check a profuse hemorrhage from a vulvar tear, involving the bulb of the vestibule. Persulphate of iron and packing had been resorted to in lieu of a few well-placed suture-ligatures, and the patient was wellnigh exsanguined. In attendance were a surgeon and obstetrician, both of marked prominence in their respective lines. Perturbed by the social prominence of their patient, however, worn out by fatigue, and rattled by anxiety and the frantic appeals of relatives, they had acted contrary to their ordinarily good judgment, and were utterly discredited. Again, it was my fortune to be present at a successful abdominal operation which revealed a ruptured uterus and the absence of several feet of ileum. Through the uterine tear several coils of the intestine had been drawn down and removed with a curet, the physician laboring under the delusion that he was dealing with placental tissues.

Paramount among the amenities incident to the practice of obstetrics are the physician's personal comfort, the appreciation of his employers and the honorarium which rewards his efforts in their behalf. A capable, attentive nurse greatly lightens one's burden. She tides the patient over her earlier trials and calls upon the medical attendant only when his presence is needed. In very truth she is a blessing! Invoke her aid always when the circumstances of your patient render possible such a luxury. Cleanly and attractive surroundings, too, certainly contribute much. Not only is asepsis rendered easier and more certain; but the reflection that a clean bed, or couch, awaits his use when rest is permissible, adds much to the doctor's satisfaction. Repose, undisturbed by even the suspicion of lurking parasitic foes, is a powerful reviver. Fresh in mind and body, with courage, judgment and vigor restored, the battle is renewed; and "the victory of endurance born" seeks to alight upon his banner.

As our patrons rise in the intellectual, social and financial scale, service in behalf of the child-bearing is more adequately appreciated and rewarded. But, mark you, this rule has many exceptions, from the enumeration of which we purposely forbear. Rather let us proceed to the principal theme, anent the fees of the obstetrician. Laconically expressed, skilled assistance deserves the highest obtainable reward; blundering incompetence should be penalized.

The average honorarium of the obstetrician, however, is grossly inadequate. For a simple case of confinement, involving little loss of time and few subsequent visits, the physician's charge should equal that of the surgeon who is called upon to amputate the arm of a member of the same family. A difficult, time-consuming labor,—one demanding the highest skill and ripest judgment, should, when brought to a successful termination, be rewarded as liberally as any major surgical operation would be. While, at first hearing, these may be regarded as the views of a radical, and quite unworthy of acquiescence, we feel convinced that reflection will establish their practical truth.

Reform in the matter of fees, such as has been suggested, would be productive of great good. Study of the science and art of obstetrics would receive a much-needed stimulus, and specialism would be encouraged. From this increased proficiency there would inevitably follow fewer traumatisms, with all their disastrous train of consequences. Mothers and babies would no longer be sacrificed unnecessarily, and in countless thousands, upon the altar of incompetence, as they now are.

But far above the enhanced reward, in real value, would be the approving sense of personal fitness. The ability to do the right thing, at the right time, and in the right manner would no longer distinguish comparatively few obstetricians from their fellows; it would glorify the whole class.

164 Franklin Street.

